# Ulnar Neuropathy Hydrodissection With Platelet Lysate and Prolotherapy: A Case Series and Review of the Literature

**DOI:** 10.7759/cureus.79791

**Published:** 2025-02-27

**Authors:** Nicholas R Hooper, Walter I Sussman, Robert Bowers, Christopher Williams

**Affiliations:** 1 Department of Physical Medicine and Rehabilitation, Emory University, Atlanta, USA; 2 Physical Medicine and Rehabilitation, Tufts Medical Center, Wellesley, USA; 3 Physical Medicine and Rehabilitation, Interventional Orthopedics of Atlanta, Atlanta, USA; 4 Physical Medicine and Rehabilitation, Regenexx Cayman, Cayman Islands, CYM

**Keywords:** cubital tunnel syndrome, nerve hydrodissection, neuroprolotherapy, orthobiologics, platelet lysate (pl), ulnar neuropathy

## Abstract

This case series highlights both pain and function outcomes of three patients who underwent hydrodissection of the ulnar nerve with platelet lysate and prolotherapy for symptomatic ulnar nerve entrapment of the elbow. All patients reported significant long-term symptom improvement, which reached more than 75% regarding pain and function and greater than 80% Single Assessment Numeric Evaluation (SANE) evaluation at the follow-up. The outcomes of this case series suggest that hydrodissection with platelet lysate and dextrose neuroprolotherapy may provide an alternative viable non-surgical treatment option for patients with ulnar neuropathy.

## Introduction

Chronic ulnar neuropathy is a common focal upper extremity compressive neuropathy [1]. Although the elbow is the most common site of entrapment, the ulnar nerve is also susceptible to compression at the forearm and wrist. Treatment may be conservative or surgical with the goal of reducing or eliminating external compression on the nerve [1,2]. Patients with mild symptoms often respond to conservative treatments (i.e., education, elbow splints, physical therapy, and nerve gliding exercises), with symptomatic relief reported in up to 90% of cases [2,3]. In patients with persistent pain, paresthesia, and signs of objective motor weakness or muscular atrophy, surgical options include decompression with or without ulnar nerve transposition [1, 2]. However, for some patients, there is a "treatment gap," where traditional conservative measures have failed to provide adequate relief or improved function, but surgery is either not an option or not worth the risk.

In this case series, we present a series of patients with ulnar neuropathy of the elbow with recurrent symptoms that were managed successfully with an ultrasound-guided hydrodissection using platelet lysate and dextrose neuroprolotherapy. Hydrodissection has previously been shown to be an effective, minimally invasive therapy in treating compression neuropathies in both the upper and lower extremities [4,5]. Nerve hydrodissection involves an injection of a solution under pressure between perineural tissue planes in order to separate fascia and release adhesions from an entrapped or compressed peripheral nerve.

Case reports have been published using various hydrodissection techniques and solutions, including anesthetic solutions with saline, dextrose, corticosteroid, platelet-rich plasma, and platelet lysate under ultrasound guidance [4,5]. Various treatments with orthobiologics have been used to improve the efficacy of hydrodissection procedures, and platelet lysate is created by lysing platelets for the removal of the platelet membrane and other cellular debris, resulting in a solution rich in growth factors, cytokines, and chemokines [6]. The combination of an anesthetic-based dextrose prolotherapy solution due to the known positive neurogenic effects with cytokine-rich platelet lysate was used for this case series with the intention of promoting both nerve axonal regrowth as well as decompressing the ulnar nerve at its site of entrapment [6]. The aim of this paper is to review the current literature on hydrodissection of the ulnar nerve and present a case series highlighting the successful use of a novel orthobiologic preparation combining prolotherapy with platelet lysate for an ultrasound-guided hydrodissection of the ulnar nerve.

## Case presentation

Procedure

The procedure for all three cases given below was performed by a single physician (C.W.) with more than 10 years of experience in musculoskeletal ultrasound. The hydrodissection was performed with an injectate solution containing platelet lysate and dextrose neuroprolotherapy. The platelet lysate was prepared with an open-processing technique that has previously been described [7]. In all three cases, the platelet lysate was combined with 50% dextrose and 0.5% ropivacaine for the creation of a 5% dextrose neuroprolotherapy solution.

**Figure 1:**
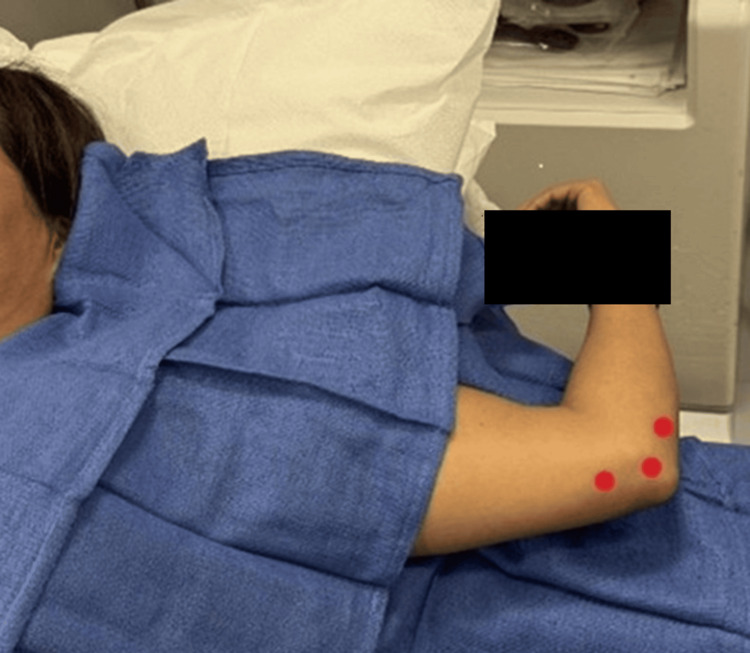
Patient positioning with three red dots corresponding to hydrodissection points at, above, and below cubital tunnel

**Figure 2:**
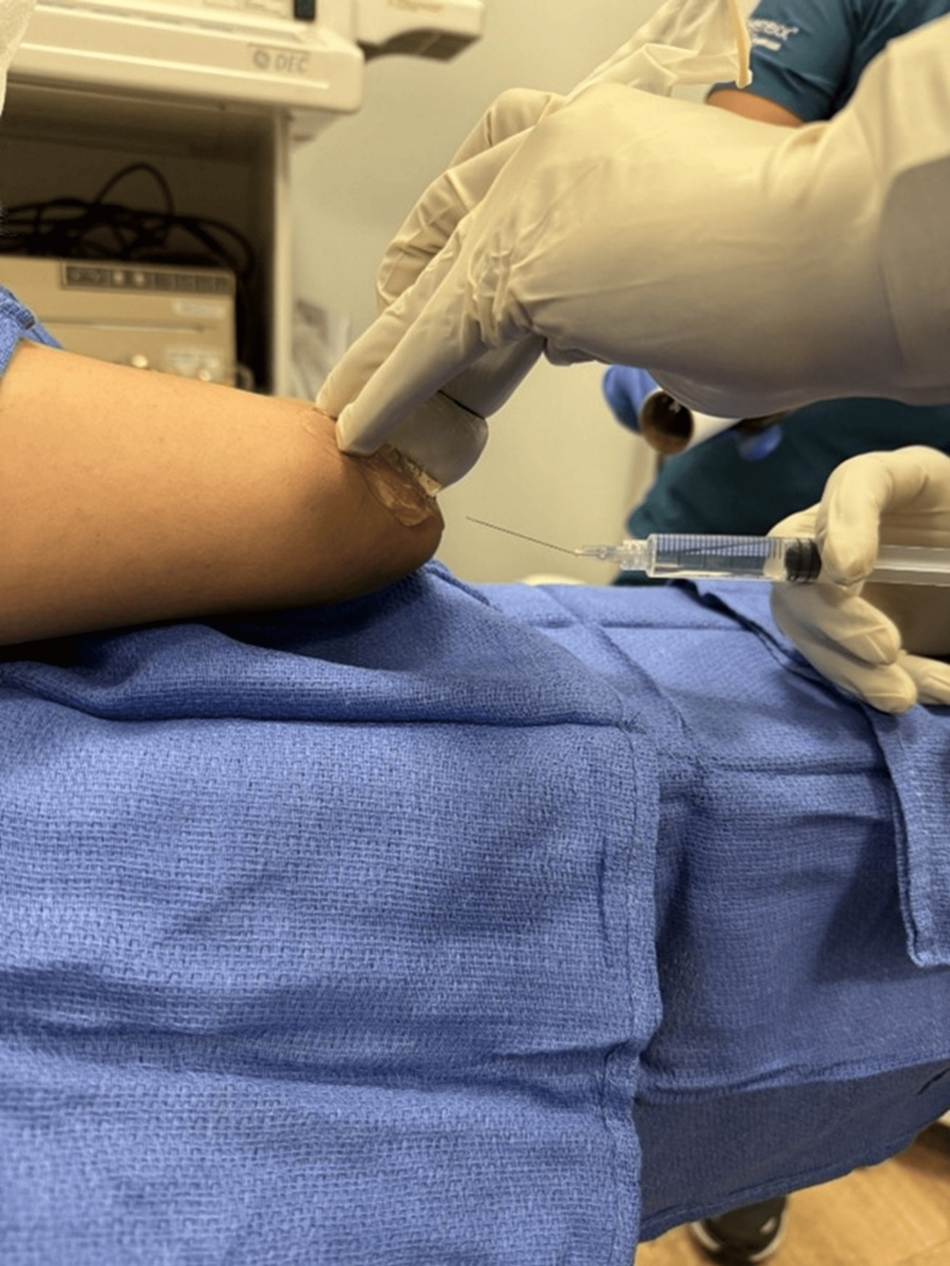
Ultrasound position and needle entry

**Figure 3:**
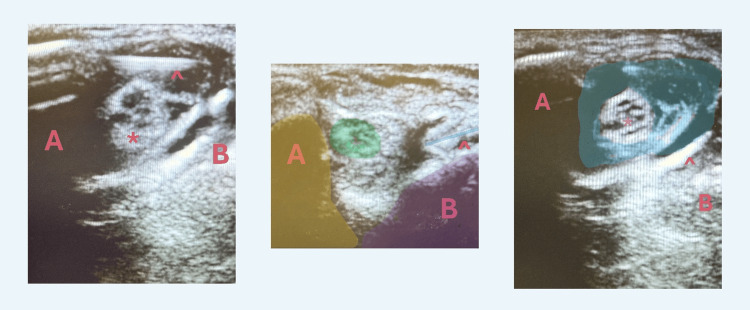
Ultrasound image demonstrating hydrodissection around ulnar nerve (A) medial epicondyle, (B) olecranon, (*) ulnar nerve, (^) needle, blue highlight =  injectate demonstrating hydrodissection around nerve

The perineural hydrodissection was performed with the patient's elbow abducted and flexed while the area was sterilized and draped (Figure 1). The entire procedure was performed using a 6-15 MHz linear array transducer (Sonosite, HFL50xp, X-Porte) and a 27-guage 1.5-inch needle with an in-plane technique short-axis to the ulnar nerve (Figure 2). The nerve was hydrodissected with equal aliquots of volume at three locations: 1) 1 cm proximal to the cubital tunnel, 2) at the cubital tunnel, and 3) and 1 cm distal to the cubital tunnel (Figure 3).

Case 1

A 31-year-old male competitive bodybuilder/powerlifter presented with chronic progressive bilateral elbow pain. He stated that one morning, he awoke from sleep and was transiently unable to flex his elbows. The pain was localized to his triceps insertion, but he also has referred left-hand dysesthesias in the ulnar nerve distribution at elbow flexion. His baseline Visual Analog Score (VAS) was 6/10, and his symptoms were worse in the mornings and while pushing heavy elements.

On initial physical exam, manual muscle testing was normal bilaterally. Sensory examination with a Wartenberg pinwheel was grossly normal from C3 to T1. Spurling testing was negative. He had significant tenderness to palpation of left triceps insertion at the olecranon as well as mild tenderness to the right common extensor tendon. He had a positive Tinel’s and positive elbow flexion testing of the left ulnar nerve. A diagnostic ultrasound was performed which revealed thickening of the ulnar nerve with a cross-sectional area (CSA) measuring 12.43 mm^2^ on the left and 7.18 mm^2^ on the right without subluxation on dynamic testing. Previous literature has suggested that an ulnar nerve CSA greater than 10 mm^2^ is indicative of pathology [8,9]. Given his symptoms of ulnar neuropathy and that the CSA of the ulnar nerve was diagnostic, an electrodiagnostic/nerve conduction study (EMG/NCS) was deferred.

The platelet lysate solution was prepared using 60 mL whole blood concentrated to 2 mL of platelet lysate for the perineural injection, combined with 0.5 mL of 50% dextrose and 2.5 mL of 0.5% ropivacaine. Additionally, the left distal triceps tendon was injected under ultrasound guidance at the olecranon insertion with a total of 4 ml of platelet-rich plasma (PRP) prepared at the same time created from an additional 60 mL whole blood draw. The patient tolerated the procedure without complications. At the two-month follow-up, the patient reported a 70% improvement in his symptoms and had returned to weightlifting. At 18 months, he had complete relief of both his neuropathic and nociceptive pain (VAS = 0/10) with the exception of occasional mild discomfort in his left triceps insertion region at the olecranon with prolonged elbow flexion. His reported Single Assessment Numeric Evaluation (SANE) at 18 months was 95%.

Case 2

A 54-year-old female presented with ongoing right elbow pain and numbness in the right third and fifth digits. She noted her pain was significantly worse at night. Her past medical history included chronic cervical pain after involvement in a motor vehicle accident 18 months prior, with elbow pain and dysesthesias starting right after the accident. Her baseline VAS score was 6/10.

On manual muscle testing, she had normal bilateral upper extremity strength with the exception of her right finger flexors, which was 4-/5. Bilateral grip strength with the hand dynamometer was 20.2 pounds on the right and 43.8 pounds on the left. She had no notable atrophy on inspection. Sensory examination was normal in the C5-T1 distribution, but examination did reveal a positive right Tinel's at the elbow.

The platelet lysate solution was made by processing 25 mL of whole blood concentrated to 2 mL of solution and combined with 0.5 mL of 50% dextrose and 2.5 mL of 0.5% ropivacaine. The patient tolerated the procedure without any complications. At her 22-month follow-up, she reported complete resolution of her neuropathic and nociceptive pain (VAS = 0/10), and her SANE was 100%.

Case 3

A 32-year-old female presented with worsening left elbow pain. She stated the pain started acutely two months prior while doing leg raises on her elbows in a plank position. Early symptoms included tingling in the left fourth and fifth digits, but over time, the neuropathic pain intensified and became constant. In addition to dysesthesias, she started to experience cramping and a crawling sensation in her hands. On average, her VAS was 4/10.

Clinical examination revealed 5/5 strength in her upper extremities except for mildly decreased left finger flexion, which was a 4+/5. On sensory exam, she had slightly decreased sensation to light touch in the C8-T1 dermatome. Provocative testing was notable for a positive Froment sign. She had pain with prolonged deep elbow flexion as well as with a positive Tinel's sign at the elbow. A diagnostic ultrasound revealed an ulnar nerve CSA of 18.1 mm^2^ at the cubital tunnel without evidence of subluxation. The symptoms and ultrasound were considered diagnostic, and an EMG/NCS was deferred.

A 115 mL whole blood draw was used to create a 4 mL solution of platelet lysate that was combined with 1 mL of 50% dextrose and 4 mL of 0.5% ropivacaine. The patient tolerated the procedure well without complications. Post-procedure, she started physiotherapy for nerve glide exercises, as well as utilization of a nighttime elbow splint. At her six-week follow-up, she noted a significant improvement in her elbow stiffness as well as her neuropathic symptoms. Overall improvement was noted to be 60%. At a 67-month follow-up, she noted continued improvement. Her VAS improved to 1/10, and she reported minor numbness with very prolonged elbow flexion, and a SANE was 80%.

## Discussion

Hydrodissection has emerged as a novel procedure for recalcitrant ulnar neuropathy [4,5]. This case series describes the use of platelet lysate and prolotherapy solution for the hydrodissection procedure. All three patients in this case series reported long-term improvement in their symptoms, and specifically, each patient reported over 75% improvement in pain and function with greater than 80% SANE at the last follow-up (Table 1). No studies have examined the minimal clinically important difference (MCID) for patients with ulnar neuropathy treated with a hydrodissection procedure, but a study of 666 patients who underwent upper extremity/hand surgery (including cubital tunnel release) found that the threshold for the MCID on a VAS was between 1.6 to 1.9 [10]. In this case series, all three of our patients met the MCID 1.9 cutoff. 

**Table 1:** Patient demographics, diagnosis and outcomes VAS- Visual Analog Scale; SANE- Single Assessment Numeric Evaluation; D5W- 5% dextrose water.

Patient	Age	Sex	Diagnosis		Hydrodissection Medication	Baseline VAS		Follow Up VAS	Follow Up SANE	Follow up period (in months)
Patient 1	31	M	Cubital Tunnel Syndrome	Platelet Lysate and D5W			6	0	95%	18
Patient 2	54	F	Cubital Tunnel Syndrome	Platelet Lysate and D5W			6	0	100%	22
Patient 3	32	F	Cubital Tunnel Syndrome	Platelet Lysate and D5W			4	1	80%	67

The use of ultrasound-guided hydrodissection of peripheral nerve compression syndromes offers patients a less invasive option than traditional surgical intervention. Although there have been multiple randomized controlled trials and large cohort studies examining the efficacy of nerve hydrodissection on carpal tunnel syndrome, studies that have evaluated its use for ulnar neuropathy have been mainly limited to case reports. Fluoroscopic-guided PRP injections around the medial elbow have been associated with injury of the ulnar nerve [11], but ultrasound-guided injections have the advantage of real-time guidance, and in reported cases of ultrasound-guided hydrodissection of the ulnar nerve, there have been no significant adverse events reported (Table 2). Cadaveric studies have suggested ultrasound-guided perineural injections are accurate and can help to avoid iatrogenic injection of the nerve or adjacent vascular structures [12,13].

**Table 2:** Outline of previous studies examining the treatment of ulnar neuropathy with hydrodissection VAS- Visual Analog Scale; DASH- Disabilities of the Arm, Shoulder, and Hand questionnaire; CSA- Cross sectional area; ESS- electrophysiological severity scale; SQUNE- self-administered questionnaire of the ulnar neuropathy at the elbow; MGS- McGowan classification; D5W- 5% dextrose water.

Study (Author, Year)	Study Design	Diagnosis	Treatment	Participant characteristics			Follow-up interval	Efficacy outcome	Results
				Number of participants	Mean age (years)	Male (%)			
Gerard 3rd et al., 2022 [14]	Case Report	Ulnar Neuropathy at the Elbow	Hydrodissection with saline, betamethasone, and lidocaine	1	19	100	7 months	Return to play, symptom reduction	Complete resolution of symptoms and returned to pitching at 7 months
Choi et al., 2015 [15]	Prospective Cohort	Cubital Tunnel Syndrome	Hydrodissection with lidocaine and triamcinolone	10	63.1	70	1 and 4 weeks	VAS, MGS, SQUNE, CSA, ESS	Significantly improved VAS and CSA at 1 and 4 weeks. Significantly improved ESS at week 4
Chen et al., 2020 [16]	Randomized Control Trial	Ulnar Neuropathy at the Elbow	Hydrodissection with either 1) D5W, or 2) Triamcinolone with normal saline	D5W-17 CS-16	D5W-55.5 CS-5635	D5W-29 CS- 37.5	1, 3, and 6 months	VAS, DASH, CSA	Significant improvements in VAS, DASH and CSA at all time points by both groups
Azza et al., 2022 [17]	Case Report	Ulnar nerve entrapment at arcade of struthers	Hydrodissection with lidocaine and triamcinolone	1	39	100	1 week	VAS, symptoms	Complete resolution of symptoms and pain
Tranchitella et al., 2024 [18]	Case Report (Video)	Ulnar Neuropathy at the Elbow	Hydrodissection with lidocaine and dexamethasone	1	41	0	3 weeks	Symptoms	“Slight, but temporary, reduction in symptoms”
Stoddard et al., 2019 [19]	Case Report	Ulnar Neuropathy at the Elbow	Hydrodissection with D5W	1	15	0	72 h; 1, 2, 3, 5 months	Pain and paresthesia	Complete resolution of paresthesia at 1 month, persistence but improvement of medial elbow pain through 5 months

In a prospective cohort, Choi et al. (2015) prospectively evaluated the effectiveness of ultrasound-guided hydrodissection of lidocaine and triamcinolone on 10 patients diagnosed with cubital tunnel syndrome [15]. The patients in this study experienced decreased pain, anatomic improvement in the ulnar nerve cross-sectional area, and electrophysiologic improvements. In addition, there have been multiple case reports showing the efficacy and safety of ulnar nerve hydrodissection at the elbow or forearm [14,17-19]. Only a single randomized controlled trial has been conducted to assess the comparative effects of different injectates for hydrodissection of ulnar neuropathy at the elbow [16]. In this study, 36 patients were either injected with 30 mg of triamcinolone or 5 ml of 5% dextrose water (D5W), and both injectates provided a significant reduction in symptoms and improved cross-sectional area of the nerve without recurrent symptoms over the six-month trial [16]. In addition, there have been multiple studies evaluating the efficacy of orthobiologics for hydrodissection of the median nerve at the wrist (carpal tunnel syndrome or CTS). One systematic review analyzed nine randomized controlled trials and found three studies that examined D5W, PRP, corticosteroids, and hyaluronidase as injectables [20]. In this systematic review, Buntragulpoontawee et al. found that both D5W and PRP consistently showed favorable outcomes when compared to the other injectates [20]. In a different review, Lee et al. completed a network meta-analysis and similarly found that D5W was the most effective in the short term and PRP more effective in the long term [21]. However, at the time of this publication, there have been no studies examining the efficacy of PRP or any platelet derivative products (e.g., platelet lysate, platelet-derived growth factor, or platelet releasate) in combination with prolotherapy for ulnar neuropathy at the elbow. 

Platelet-derived orthobiologics are derived from autologous whole blood that is centrifuged to form an injectate that has a higher platelet concentration than whole blood and is rich in cytokines and growth factors, such as the nerve growth factor (NGF), brain-derived neurotrophic factor (BDNF), vascular endothelial growth factor (VEGF), and transforming growth factor (TGF-β) that are crucial for promoting axonal nerve regrowth [6,22,23]. These cytokines and growth factors are thought to promote axonal regrowth and potentially restore the target nerve function [24,25]. Platelet lysate undergoes an additional processing step to lyse the platelets and produce a cell-free supernatant [6,26,27].

Different techniques have been described to produce platelet lysate, but the freeze-thaw method described by Centeno et al. was used in these cases [7]. It is thought that this additional step also lyses leukocytes and produces a product that lacks white blood cells (WBC). Leukocytes release proteases and other constituents that cause increased pro-inflammatory effects [28,29], and platelet lysate has been used for peripheral and lumbar radiculopathy to minimize a post-injection inflammatory response [7]. A study by Centeno et al. found that patients with lumbar radicular pain reported significant improvements compared to baseline when treated with a platelet lysate epidural [7]. Based on these prior studies, the authors elected to use platelet lysate as an injectate over PRP [30].

Multiple studies have also examined the efficacy of prolotherapy for peripheral entrapment neuropathy. Prolotherapy has been proposed to bind to presynaptic calcium channels and inhibit substance-P and calcitonin gene-related peptides, which leads to decreased neurogenic inflammation [29]. Wu et al. found that when compared to saline control, patients with carpal tunnel syndrome experienced significant relief with perineural injection of D5W [31]. A randomized control trial (RCT) examining the efficacy of PRP versus dextrose prolotherapy for CTS by Shen et al. reported that the PRP group experienced a significantly greater reduction in symptoms at three months but not at six months [32]. However, the PRP group had a significant reduction in the cross-sectional area of the median nerve when compared to D5W at both three and six months [32]. 

The mechanism for the clinical improvement in the cases presented here remains unclear. The effects of the injectate volume during the nerve hydrodissection are designed to separate tissue planes and release adhesions around the perineural region. In addition, it has been postulated that the volume of the injection displaces local inflammatory and noxious cytokines that may also result in a therapeutic benefit [33]. Previous studies have shown improvements in pain with both the use of neural prolotherapy and platelet-derived orthobiologics, and both injectates were used for these reported hydrodissection procedures.

Despite limitations involving the mechanism of action, this case series aimed to provide clinical examples of long-term improvement in ulnar neuropathy symptoms following ulnar nerve hydrodissection with platelet lysate and prolotherapy. To the authors' knowledge, this combination of injectates has not been reported yet in the literature. Limitations include those inherent in a case series, including variations in presentation, inconsistent preparation of injectate, multiple injectates utilized, and variable injection approaches. Despite these limitations, this case series provides clinical examples regarding the long-term effectiveness and safety of ulnar nerve hydrodissection utilizing a neuroprolotherapy solution with platelet lysate. Future randomized control trials directly comparing platelet lysate to other common injectates are needed.

## Conclusions

To the best of our knowledge, this is the first case series to report the use of combined platelet lysate and prolotherapy for the hydrodissection of the ulnar nerve. This technique resulted in the resolution or improvement of all three patient's neuropathy symptoms. While future research is warranted, these cases suggest that hydrodissection with platelet lysate and dextrose neuroprolotherapy may provide a viable non-surgical treatment option in patients with ulnar neuropathy.
